# Author Correction: Variants in *SART3* cause a spliceosomopathy characterised by failure of testis development and neuronal defects

**DOI:** 10.1038/s41467-023-39372-x

**Published:** 2023-06-15

**Authors:** Katie L. Ayers, Stefanie Eggers, Ben N. Rollo, Katherine R. Smith, Nadia M. Davidson, Nicole A. Siddall, Liang Zhao, Josephine Bowles, Karin Weiss, Ginevra Zanni, Lydie Burglen, Shay Ben-Shachar, Jenny Rosensaft, Annick Raas-Rothschild, Anne Jørgensen, Ralf B. Schittenhelm, Cheng Huang, Gorjana Robevska, Jocelyn van den Bergen, Franca Casagranda, Justyna Cyza, Svenja Pachernegg, David K. Wright, Melanie Bahlo, Alicia Oshlack, Terrence J. O’Brien, Patrick Kwan, Peter Koopman, Gary R. Hime, Nadine Girard, Chen Hoffmann, Yuval Shilon, Amnon Zung, Enrico Bertini, Mathieu Milh, Bochra Ben Rhouma, Neila Belguith, Anu Bashamboo, Kenneth McElreavey, Ehud Banne, Naomi Weintrob, Bruria BenZeev, Andrew H. Sinclair

**Affiliations:** 1 The Murdoch Children’s Research Institute, Melbourne, VIC Australia; 2 Department of Paediatrics, The University of Melbourne, Melbourne, VIC Australia; 3 The Victorian Clinical Genetics Services, Melbourne, VIC Australia; 4 Department of Neuroscience, Central Clinical School, Monash University, Alfred Centre, Melbourne, VIC Australia; 5 Walter and Eliza Hall Institute of Medical Research, Melbourne, VIC Australia; 6 School of BioSciences, Faculty of Science, University of Melbourne, Melbourne, VIC Australia; 7 Department of Medical Biology, Faculty of Medicine, Dentistry and Health Sciences, University of Melbourne, Melbourne, VIC Australia; 8 Department of Anatomy and Physiology, The University of Melbourne, Melbourne, VIC Australia; 9 Institute for Molecular Bioscience, The University of Queensland, Brisbane, QLD Australia; 10 School of Biomedical Sciences, The University of Queensland, Brisbane, QLD Australia; 11 Genetics Institute, Rambam Health Care Campus, Rappaport Faculty of Medicine, Institute of Technology, Haifa, Israel; 12 Unit of Muscular and Neurodegenerative Disorders and Unit of Developmental Neurology, Department of Neurosciences, Bambino Gesù Children’s Hospital, IRCCS, Rome, Italy; 13 Centre de Référence des Malformations et Maladies Congénitales du Cervelet, Et Laboratoire de Neurogénétique Moléculaire, Département de Génétique et Embryologie Médicale, APHP. Sorbonne Université, Hôpital Trousseau, Paris, France; 14 Developmental Brain Disorders Laboratory, Imagine Institute, INSERM UMR 1163 Paris, France; 15 Genetic Institute, Tel Aviv Sourasky Medical Center, Tel Aviv, Israel; 16 Genetics Institute, Kaplan Medical Center, Hebrew University Hadassah Medical School, Rehovot, 76100 Israel; 17 Edmond and Lily Safra Children’s Hospital, Chaim Sheba Medical Center, Ramat Gan, Israel; 18 Sackler School of Medicine, Tel Aviv University, Tel Aviv, Israel; 19 Department of Growth and Reproduction, Copenhagen University Hospital, Rigshospitalet, Copenhagen, Denmark; 20 Monash Proteomics and Metabolomics Facility, Biomedicine Discovery Institute, Department of Biochemistry and Molecular Biology, Monash University, Clayton, VIC Australia; 21 The Peter MacCallum Cancer Centre, Melbourne, VIC Australia; 22 School of Mathematics and Statistics, The University of Melbourne, Melbourne, VIC Australia; 23 Department of Medicine, The Royal Melbourne Hospital, The University of Melbourne, Melbourne, VIC Australia; 24 Department of Pediatric Neurology, Aix-Marseille Université, APHM, Timone Hospital, Marseille, France; 25 Radiology Department, Sheba medical Centre, Tel Aviv, Israel; 26 Kaplan Medical Center, Hebrew University Hadassah Medical School, Rehovot, 76100 Israel; 27 Pediatrics Department, Kaplan Medical Center, Rehovot, 76100 Israel; 28 Faculty of Medicine, Hebrew University of Jerusalem, Hadassah Medical School, Jerusalem, Israel; 29 Higher Institute of Nursing Sciences of Gabes, University of Gabes, Gabes, Tunisia; 30 Laboratory of Human Molecular Genetics, Faculty of Medicine of Sfax, Sfax University, Sfax, Tunisia; 31 Department of Congenital and Hereditary Diseases, Charles Nicolle Hospital, Tunis, Tunisia; 32 Institut Pasteur, Université de Paris, CNRS UMR3738, Human Developmental Genetics, 75015 Paris, France; 33 The Rina Mor Genetic Institute, Wolfson Medical Center, Holon, 58100 Israel; 34 Pediatric Endocrinology Unit, Dana-Dwek Children’s Hospital, Tel Aviv Medical Center, Tel Aviv, Israel; 35 Sheba Medical Center, Tel Aviv, Israel; 36 Present Address: Department of Medical Biology, Faculty of Medicine, Dentistry and Health Sciences, University of Melbourne, Melbourne, VIC Australia

**Keywords:** Disease genetics, Genetics research, Disease model, Gonadal disorders, Neurodevelopmental disorders

Correction to: *Nature Communications* 10.1038/s41467-023-39040-0, published online 09 June 2023

In this article, the author name Kenneth McElreavey was incorrectly written as Kenneth MacElreavey.

The original article has been corrected.

