# Supplementary material for: Variants in SART3 cause a spliceosomopathy characterised by failure of testis development and neuronal defects
Source: Nat Commun. 2023 Jun 9;14:3403. doi: 10.1038/s41467-023-39040-0 (PMC10256788; doi:10.1038/s41467-023-39040-0)
Supplement: Supplementary file 1 — Supplementary Information [file 41467_2023_39040_MOESM1_ESM.pdf]

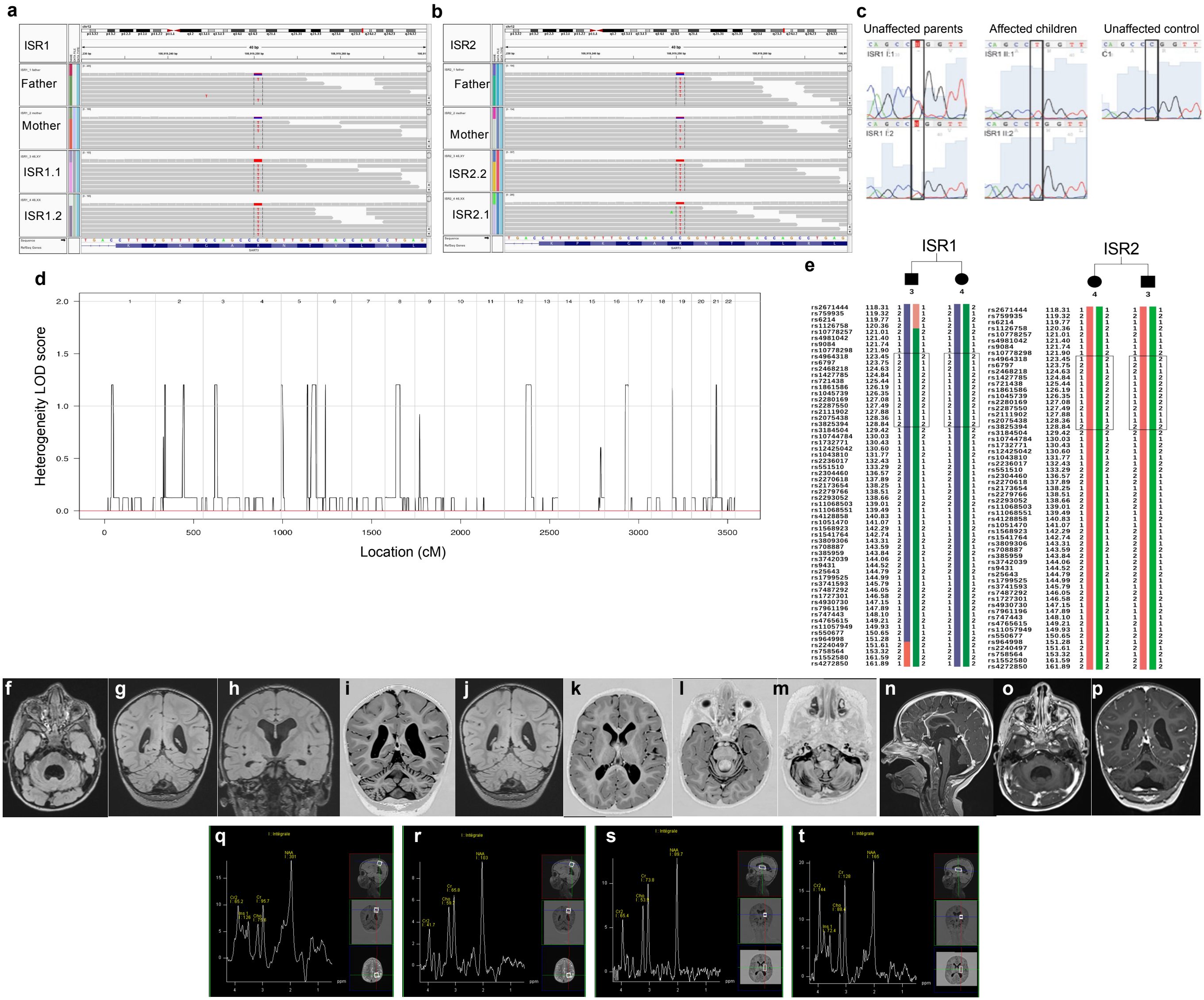

a
ISR1
Father
Mother
ISR1.1
ISR1.2
b
ISR2
Father
Mother
ISR2.2
ISR2.1
c
Unaffected parents
Affected children
Unaffected control
ISR1 I:1
ISR1 I:2
ISR1 II:1
ISR1 II:2
C1
d
Heterogeneity LOD score
Location (cM)
e
ISR1
ISR2
f
g
h
i
j
k
l
m
n
o
p
q
r
s
t
I : Intégrale
NAA
Cr
Cho
Cr2
Ins 1
ppm

Supplementary Figure 1. Rare variant in *SART3* with recessive inheritance cause a novel disorder. (a,b) Snapshot from Integrated Genomics Viewer (IGV) showing the *SART3*; NM_014706.3: c.2507G>A: p.Arg836Gln in families ISR1 and ISR2. The variant is homozygous in all four affected children and heterozygous in all four unaffected parents. (c) Sanger sequencing confirmation. (d) Parametric multipoint linkage analysis. Genome-wide heterogeneity LOD scores. The maximum hLOD score of 1.2 is achieved by twelve peaks located on nine chromosomes which correspond to locations where each sibling pair are inferred to share two haplotypes identical by descent. (e) Inferred haplotypes in the region of the chromosome 12 linkage peak where the longest run of common genotypes is found. Each pair of siblings share two haplotypes identically by descent. The four cases share identical genotypes and haplotype sequences for a stretch of 12 alleles from 123.45 cM to 128.84 cM (boxed). Inspection of the set of all informative SNPs homozygosity for 47 consecutive SNPs extending over a 1.49 cM region. The *SART3* variant falls within this region. (f-p) Additional imaging and analysis for FRA1.1. (f-j) Axial and coronal flair images. (j) Coronal T1 inversion recovery (0.9mm thick). (k) Coronal flair image (0,9 mm thick).

(l-m) Axial T1 images inversion recovery (0.9mm thick). (n-p) Post gadolinium sagittal axial and coronal T1 images. (q-t) Magnetic resonance spectroscopy. White matter choline is low.

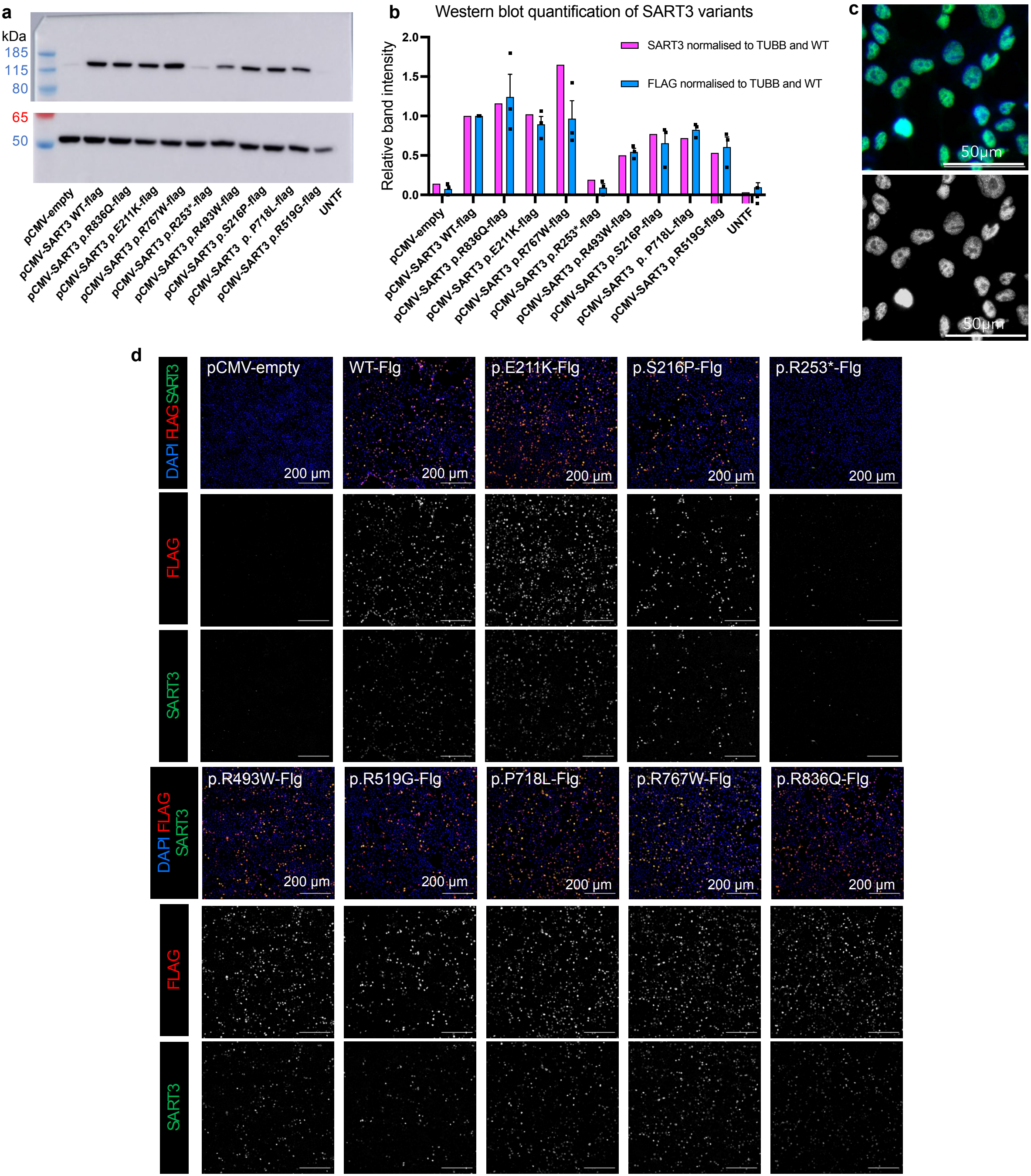

a
kDa
185
115
80
65
50
pCMV-empty
pCMV-SART3 WT-flag
pCMV-SART3 p.R836Q-flag
pCMV-SART3 p.E211K-flag
pCMV-SART3 p.R767W-flag
pCMV-SART3 p.R253*-flag
pCMV-SART3 p.R493W-flag
pCMV-SART3 p.S216P-flag
pCMV-SART3 p. P718L-flag
pCMV-SART3 p.R519G-flag
UNTF
b
Western blot quantification of SART3 variants
Relative band intensity
2.0
1.5
1.0
0.5
0.0
SART3 normalised to TUBB and WT
FLAG normalised to TUBB and WT
c
50µm
50µm
d
DAPI FLAG SART3
FLAG
SART3
pCMV-empty
WT-Flg
p.E211K-Flg
p.S216P-Flg
p.R253*-Flg
p.R493W-Flg
p.R519G-Flg
p.P718L-Flg
p.R767W-Flg
p.R836Q-Flg
200 µm

Supplementary Figure 2. SART3 variant proteins. (a) Western blot analysis of SART3-FLAG (FLG) variants expressed from a pCMV-vector using an antibody to the N-terminal part of SART3. A single band just above 115kDa is observed. (b) Quantification of the SART3 band normalized to loading control (beta-Tubulin, TUBB) relative to WT using either N-terminal SART3 antibody or anti-FLAG. Data is mean ± SEM from $n = 3$ independent experiments (FLAG antibody) or $n = 1$ (SART3 antibody). (c) Endogenous SART3 staining in HEK293t cells. d. Transient transfection of HEK293t cells using pCMV-SART3-FLAG for WT and patient variants. Staining for SART3 (green) and the C-terminal FLAG (red) are shown. DAPI is in the overlay (blue). Raw data and uncropped blots are provided in the source data file.

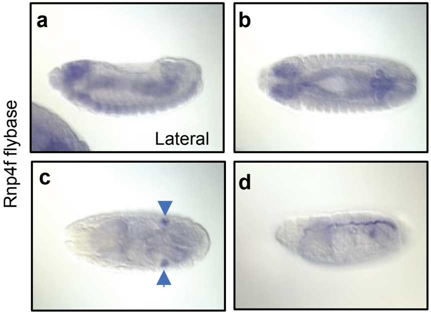

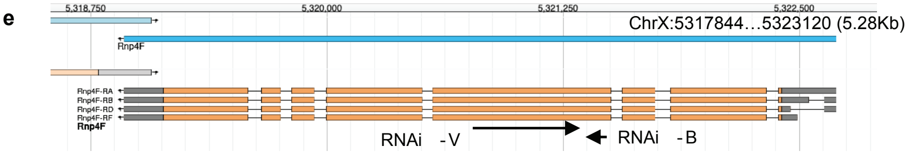

f

RNAi line	Reference	Source	Chr	KD efficiency (% KD mRNA)	Expected survival (%)	Observed survival (%)	Phenotype
RNAi-B	BL58168	Bloomington Stock Center	2	70%	25%	22/162 (13%)	Embryonic and pupal death. Adults: Held out wings, atrophic.
RNAi-V	V107063 (KK102446)	Vienna Resource Center	2	51%	50%	13/44 (29%)	Pupal death. Adults: Held out wing, atrophic, proboscis defects. Died after 2-3 days.

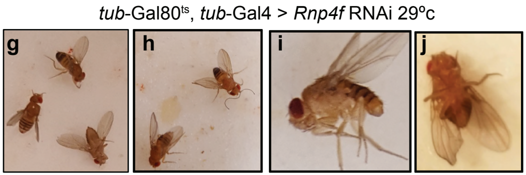

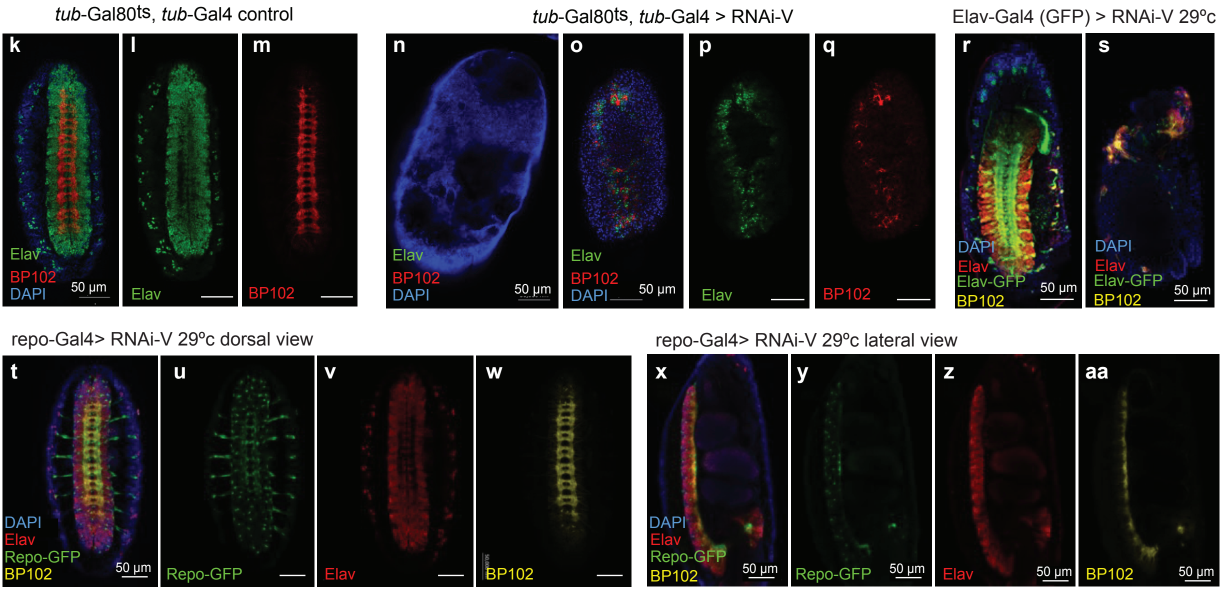

Supplementary Figure 3. *Drosophila* SART3 ortholog Rnp-4f is required for viability and neuronal development.

(a-d) *Rnp4f in situ* hybridisation on *Drosophila melanogaster* embryos (Flybase). a. stage 11-12, (b-d) stage 13-16. Strong expression is observed in the gonads (arrows) CNS and midgut[1,2,3]. e. *Rnp4f* transcripts and two RNAi lines on genome assembly BDGP Release 6 + ISO1 MT/dm6. Both target all *Rnp4f* transcripts[4,5].(f) RNAi line details. mRNA percentage KD in adults with *tub*-Gal4; t*ub*-Gal80^{ts} > RNAi at the permissive temperature as assessed by digital droplet PCR. The number of emerging adults for the correct genotype and the percentage of expected adults with the correct genotype.

Some lethality was observed for both RNAi lines at the permissive temperate of 29ºc, likely due to embryonic and pupal death. (g-j) Emerging adults had a held-out wing phenotype, and general atrophy (appeared starved) (g, i) RNAi-B, (h, j) RNAi-V. Adults with the RNAi-V line had proboscis defects and did not survive more than 2-3 days. (k-aa) Ubiquitous KD embryos stained for CNS. Control crosses (k-m) and RNAi crosses (n-q). KD embryos had significant defects (n). Some milder phenotypes were observed such as holes along the midline (o-q). Staining includes neuronal marker Elav (Embryonic lethal, abnormal vision) (green; k, l, n, o, p) and neuronal axon marker BP102 (red; k, n, m, o, q). k, n, o are overlays with DAPI (blue). (r, s) Neuronal expression of RNAi-V at 29ºc causes embryonic lethality due to CNS defects of variable severity. Elav-GFP and Elav antibody (red) marks post-mitotic neurons, BP102 marks the axons and commissures (yellow), and DAPI marks nuclei (blue). (t-aa) RNAi-V expression in the glia using the *Repo*Gal4 driver does not cause any noticeable phenotype at 29ºc. The Glia are marked with GFP (Repo, green, v and z), the neurons are marked by Elav (red, w, z). Neuronal axons are marked by BP102 highlighting the commissures (yellow, x, aa). (u-x) represent a dorsal view and (y-aa) a lateral view. (t and x) are overlays.

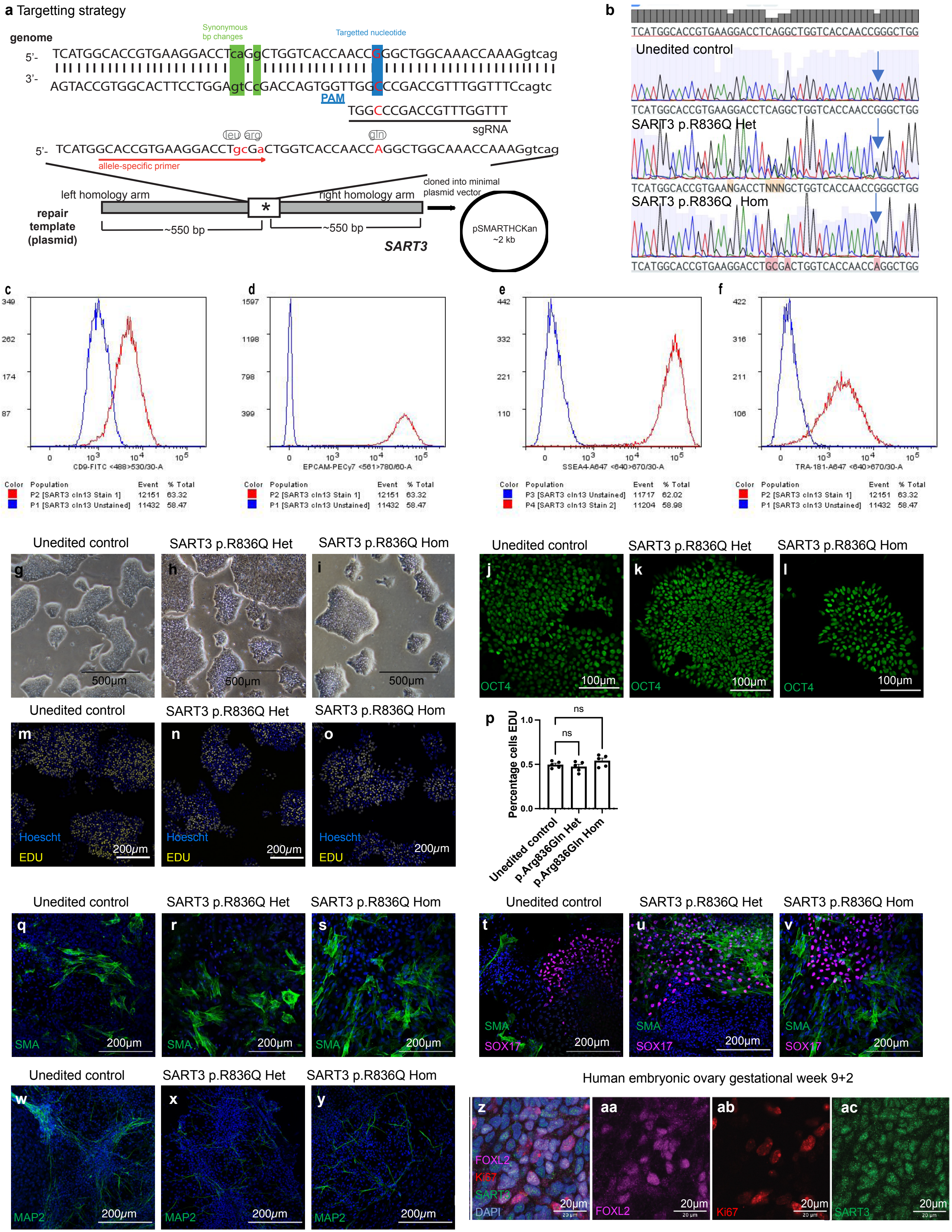

a Targetting strategy
genome
Synonymous bp changes
Targetted nucleotide
5'- TCATGGCACCGTGAAGGACCTcaGgCTGGTCACCAACCGGGCTGGCAAACCAAAGgtcag
3'- AGTACCGTGGCACTTCCTGGAgtCcGACCAGTGGTTGGCCCGACCGTTTGGTTTCcagtc
PAM
TGGCCCGACCGTTTGGTTT
sgRNA
leu arg
gln
5'- TCATGGCACCGTGAAGGACCTgcGaCTGGTCACCAACCAGGCTGGCAAACCAAAGgtcag
allele-specific primer
left homology arm
right homology arm
cloned into minimal plasmid vector
repair template (plasmid)
~550 bp
~550 bp
SART3
pSMARTHCKan ~2 kb
b
TCATGGCACCGTGAAGGACCTCAGGCTGGTCACCAACCGGGCTGG
Unedited control
TCATGGCACCGTGAAGGACCTCAGGCTGGTCACCAACCGGGCTGG
SART3 p.R836Q Het
TCATGGCACCGTGAANGACCTNNNGCTGGTCACCAACCGGGCTGG
SART3 p.R836Q Hom
TCATGGCACCGTGAAGGACCTGCGACTGGTCACCAACCAGGCTGG
c
CD9-FITC <488>530/30-A
Color Population Event % Total
P2 [SART3 cln13 Stain 1] 12151 63.32
P1 [SART3 cln13 Unstained] 11432 58.47
d
EPCAM-PECy7 <561>780/60-A
Color Population Event % Total
P2 [SART3 cln13 Stain 1] 12151 63.32
P1 [SART3 cln13 Unstained] 11432 58.47
e
SSEA4-A647 <640>670/30-A
Color Population Event % Total
P3 [SART3 cln13 Unstained] 11717 62.02
P4 [SART3 cln13 Stain 2] 11204 58.98
f
TRA-181-A647 <640>670/30-A
Color Population Event % Total
P2 [SART3 cln13 Stain 1] 12151 63.32
P1 [SART3 cln13 Unstained] 11432 58.47
Unedited control
SART3 p.R836Q Het
SART3 p.R836Q Hom
g h i
500µm
j k l
OCT4
100µm
m n o
Hoescht
EDU
200µm
p
Percentage cells EDU
ns
ns
Unedited control
p.Arg836Gln Het
p.Arg836Gln Hom
q r s
SMA
200µm
t u v
SMA
SOX17
w x y
MAP2
Human embryonic ovary gestational week 9+2
z aa ab ac
FOXL2
Ki67
SART3
DAPI
20µm

Supplementary Figure 4. Creation and validation of iPSCs carrying SART3 patient variant p.Arg836Gln. (a) The CRISPR targeting strategy to make patient variant knock-in iPSCs at the Murdoch Children's Research Institute gene editing facility, Melbourne, Australia. (b) Sanger sequencing of unedited control line, heterozygous and homozygous clones. (c-f) Confirmation of pluripotency marker expression by flow cytometry (LSR Fortessa X20) with antibodies to CD9 (c), EPCAM (d) SSEA4 (e) and TRA181 (f). Representative histograms show the antigenic phenotype of iPSCs. Red represents samples stained for specific surface antigens, whereas blue correspond to unstained control samples. (g-i) Brightfield of iPSC colonies. (j-l) OCT3/4 staining of iPSCs. (m-o. p) Click-iT EdU/Hoescht analysis of iPSCs and its quantification found no difference in proliferation rates between clones. Mean ± SEM. *P* values were calculated using a one-way ANOVA and were not significant (> 0.1). *n* = 5 wells of an 8 well chamber slide. (q-y) Embryoid bodies made from the three iPSC lines stained for antibody markers of the three germ layers. All iPSC lines were able to form embryoid bodies *in vitro*, which expressed markers consistent with the development of the three germ layers ectoderm, mesoderm and endoderm. Specifically, (q-s and t-v) Smooth Muscle Actin (SMA, green) marks the mesoderm.(t-v) SOX17 (magenta) marks the endoderm. (w-y) MAP2 (green) marks the ectoderm. DAPI is in blue. (z-ac) Week 9+2 gestation human embryonic ovaries. FOXL2 (magenta, aa) marks the granulosa cells, Ki67 marks the germ cells (ab, red). SART3 is expressed in all cells in a mostly nuclear speckled pattern (green, ac). z is an overlay with DAPI (blue).

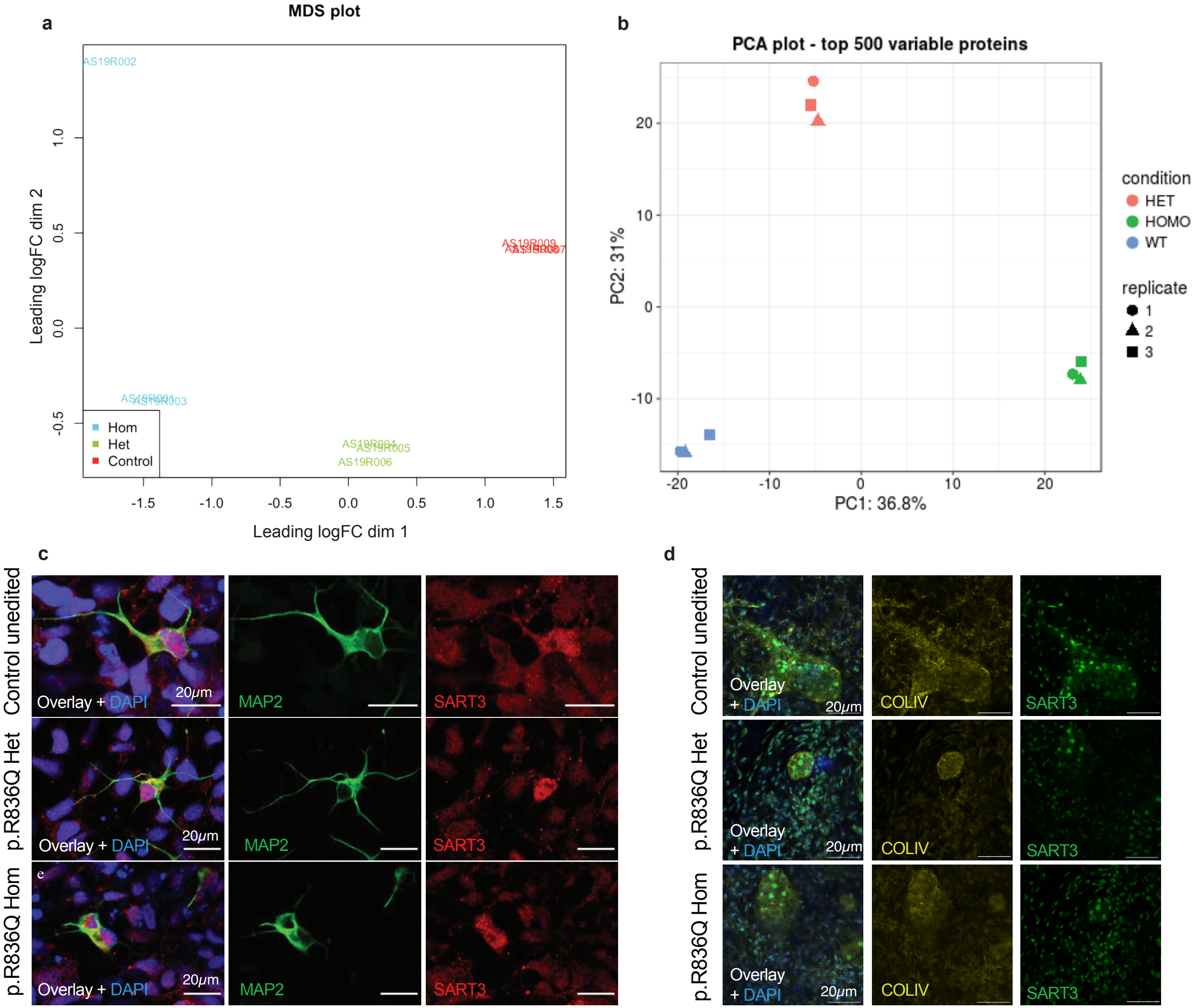
a
MDS plot
AS19R002
AS19R009
AS19R007
AS19R001
AS19R003
AS19R004
AS19R005
AS19R006
Hom
Het
Control
Leading logFC dim 2
Leading logFC dim 1
b
PCA plot - top 500 variable proteins
condition
HET
HOMO
WT
replicate
1
2
3
PC2: 31%
PC1: 36.8%
c
Control unedited
p.R836Q Het
p.R836Q Hom
Overlay + DAPI
MAP2
SART3
20μm
d
COLIV
SART3
Overlay + DAPI
20μm

Supplementary Figure 5. The SART3 p.Arg836Gln variant disrupts signalling but is not mislocalised a,b Principal component analysis (PCA) plot showing that the three biological replicates for each genotype iPSC line group together, with significant differences between genotypes in the (a) transcriptome and (b) proteome. (c) High magnification of Day 14 NGN-2 iPSC derived neurons stained for MAP2 and SART3, overlay images are shown with DAPI. SART3 appears to be both nuclear and cytoplasmic in all three lines/genotypes. (d) Day 14 gonad organoids stained for DAPI, SART3 and COLIV. SART3 shows predominantly nuclear expression, which is unchanged in the variant cells.

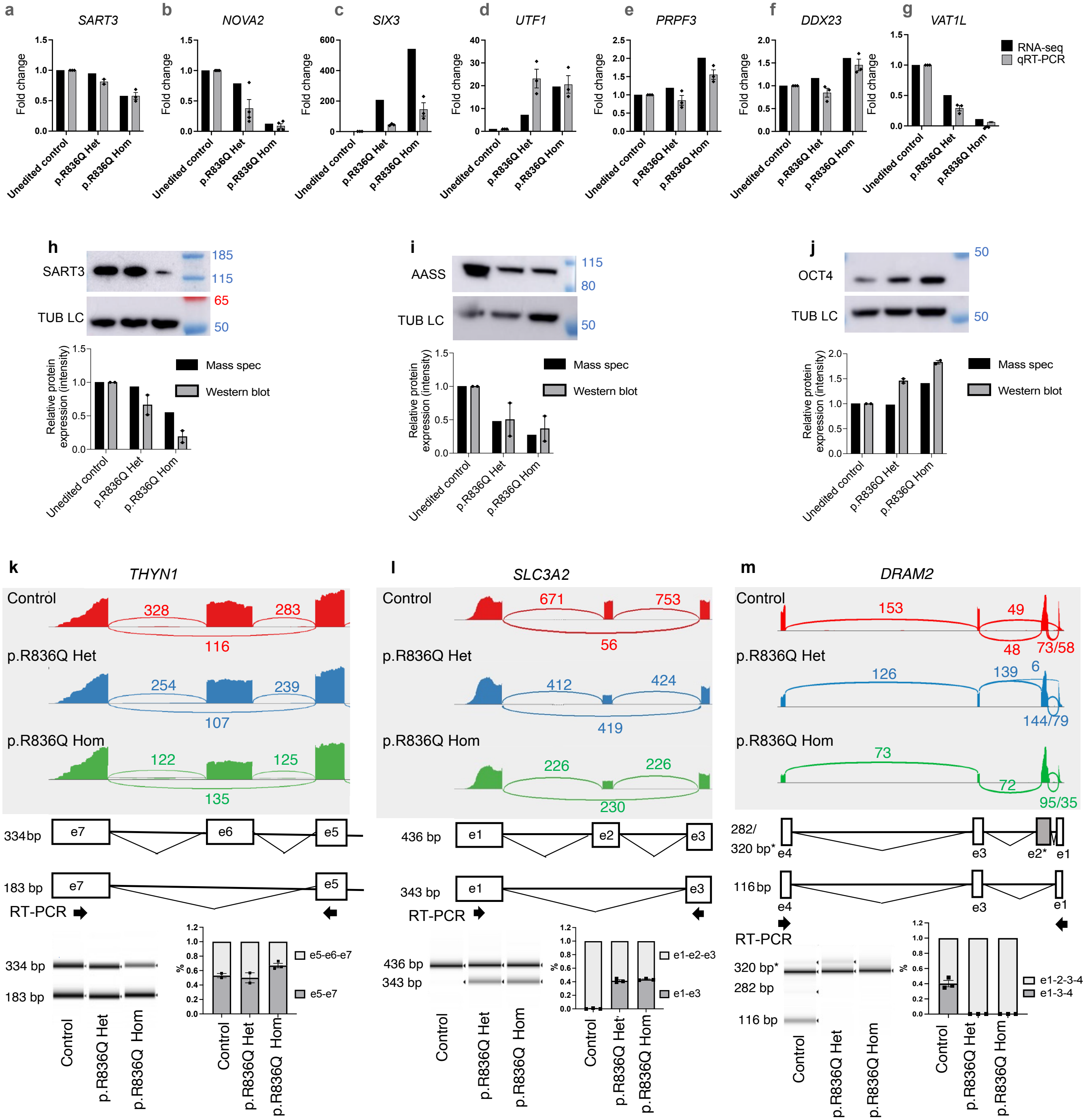

a SART3
b NOVA2
c SIX3
d UTF1
e PRPF3
f DDX23
g VAT1L
Fold change
Unedited control
p.R836Q Het
p.R836Q Hom
RNA-seq
qRT-PCR
h SART3
TUB LC
i AASS
j OCT4
Relative protein expression (intensity)
Mass spec
Western blot
k THYN1
l SLC3A2
m DRAM2
Control
p.R836Q Het
p.R836Q Hom
RT-PCR
e5-e6-e7
e5-e7
e1-e2-e3
e1-e3
e1-2-3-4
e1-3-4

Supplementary Figure 6. Validation of RNA sequencing and mass spectrometry data. (a-g) RNA-seq data and validation by qRT-PCR. Black bars represent RNA-seq data in fold change. qRT-PCR (grey bars) was carried out on three separate sets of unedited control, heterozygous and homozygous p.Arg836Gln variant iPSCs. Data is represented as mean fold change ± SEM between the *SART3* variant cells and unedited control. A one-way ANOVA followed by Dunnett's multiple comparison test was used, *P* values = * <0.05; ** <0.01; *** <0.001. (h-j) western blot validation of mass spectrometry findings for SART3 (h), AASS (i) and OCT4 (j). For each protein a representative blot is shown as well as the alpha-tubulin loading control (TUBB LC), and quantification from two separate blots with two separate iPSC sets are shown with relative protein intensity normalised to the loading control. (k-m) Validation of differential transcript usage events for three genes *THYN1* (k), *SLC3A2* (l) and *DRAM2* (m). For each, a sashimi plot from IGV showing read coverage on exons and exon junction coverage is shown, highlighting different exon usage. A schematic of exons, primers (arrows) used for RT-PCR, and amplicon size are shown. A representative image from RT-PCR migration on a Tape Station bioanalyser is included. Quantification from 2 or 3 independent sets of iPSCs are shown. Raw data and uncropped images are provided in source data file.

	ID 1	ID 2	Z0	Z1	Z2	PI_HAT
Parent child pairs	ISR2 M	ISR2 II:2	0	1	0	0.5
	ISR2 M	ISR2 II:1	0	1	0	0.5
	ISR2 F	ISR2 II:2	0	1	0	0.5
	ISR2 F	ISR2 II:1	0	1	0	0.5
	ISR1 M	ISR1 II:2	0	1	0	0.5
	ISR1 M	ISR1 II:1	0	1	0	0.5
	ISR1 F	ISR1 II:2	0	1	0	0.5
	ISR1 F	ISR1 II:1	0	1	0	0.5
Sibling pairs	ISR1 II:1	ISR1 II:2	0.211	0.691	0.098	0.443
	ISR2 II:1	ISR2 II:2	0.266	0.567	0.167	0.45
	ISR1 M	ISR2 II:2	0.565	0.406	0.029	0.232
	ISR1 II:2	ISR2 II:2	0.561	0.401	0.038	0.238
	ISR1 II:2	ISR2 F	0.56	0.395	0.046	0.243
	ISR1 II:1	ISR2 II:1	0.568	0.389	0.043	0.238
	ISR1 II:1	ISR2 II:2	0.561	0.386	0.053	0.246
	ISR1 II:2	ISR2 II:1	0.575	0.379	0.046	0.236
	ISR1 II:1	ISR2 F	0.568	0.379	0.054	0.243
	ISR1 II:1	ISR2 M	0.592	0.378	0.029	0.218
	ISR1 M	ISR2 F	0.573	0.369	0.058	0.242
	ISR1 M	ISR2 II:1	0.598	0.361	0.041	0.222
	ISR1 F	ISR2 II:1	0.62	0.346	0.034	0.207
	ISR1 F	ISR2 F	0.624	0.343	0.033	0.205
	ISR1 M	ISR2 II:2	0.632	0.338	0.03	0.199
	ISR1 F	ISR2 II:2	0.63	0.336	0.035	0.203
	ISR1 F	ISR2 M	0.649	0.332	0.019	0.185
	ISR1 11:2	ISR2 M	0.664	0.273	0.064	0.2
Co-parents	ISR2 F	ISR2 M	0.8	0.2	0	0.1
	ISR1 F	ISR2 M	0.811	0.189	0	0.094

Supplementary Table 1. PLINK analysis for ISR1 and ISR2

The proportion of alleles (PI_HAT) along with the proportion of SNPs at which each pair of individuals are estimated to share 0, 1 and 2 alleles identical by descent (IBD) between each pairwise combination of the eight sequenced individuals, as estimated by PLINK. ZN = proportion of markers at which the pair is estimated to shared N alleles inherited IBD.M = mother, F = father. This analysis validates the pedigree structures provided by indicating that each parent-child pair shares exactly one allele IBD at every SNP (parent child pairs, first eight row) and that each set of siblings shares close to the expected 50% of alleles IBD (sibling pairs, ninth and tenth rows). PLINK estimates that every one of the eight individuals is related to the other eight individuals (eleventh to twenty sixth rows). The ISR2 father is estimated to be related to co-parent ISR2 mother, while parents of ISR1 are also estimated to be related to each other (co-parents, last two rows). This suggests that haplotype sharing across the families is now more likely.

Family	Variant details				Inheritance	Variant frequencies		Cancer association	Variant in-silico predictions					
	Genomic (GRCh37)	Exon	cDNA NM_014706.3	Protein NP_055521.1		dbSNP138	GnomAD	COSMIC	Polyphen-2	PROVEAN	SNAP-2	muPRO	SIFT	GERP
ISR1	Chr12:g.108919250 C>T	17	c.2507G>A	p.Arg836Gln	Maternal/Paternal	-	0	COSV57207507 c.2506C>T p.R836W. Malignant melanoma - somatic.	1. PD	-3.09. D	64. E	-0.656. D	0.005. D	5.87
ISR2	Chr12:g.108919250 C>T	17	c.2507G>A	p.Arg836Gln	Maternal/Paternal	-	0	As above	1. PD	as above	as above	as above	as above	as above
TUN1	Chr12:g.108939013G>A	4	c.631G>A	p.Glu211Lys	Maternal/Paternal	-	0	Not present	1. PD	-3.79. D	53. E	-1.527. D	0. D	6.07
ISR3	Chr12:g.108919947G>A	16	c.2299C>T	p.Arg767Trp	Paternal	rs779659299	1 European (no homs). AF = 0.000003976	Not present	1. PD	-7.22. D	66. E	-0.898. D	0.001. D	3.91
	Chr12:g.108938227G>A	5	c.757C>T	p.Arg253*	Maternal	rs759058288	2 Europeans (no homs). AF = 0.000007953	COSV104390507 Colon Carcinoma. Somatic.	-	-	-	-	-	-
ITA1	Chr12:g.108929214C>T	12	c.1477C>T	p.Arg493Trp	Maternal	-	0	Not present	1. PD	-7.49. D	72. E	-0.698. D	0. D	5.03
	Chr12:g.12:108938998T>C	4	c.646T>C	p.Ser216Pro	Paternal	rs757404064	1 European (no homs). AF = 0.000003976	Not present	0.973 PD	-2.28. D	54. E	-1.056. D	0.055. T	4.96
FRA1	Chr12:g.108920093G>A	16	c.2153C>T	p.Pro718Leu	Maternal	-	0	Not present	1 PD	-3.88. D	3. E	-0.331. D	0.135. T	5.81
	Chr12:g.108929136T>C	12	c.1555A>G	p.Arg519Gly	Paternal	-	0	Not present	1 PD	-6.03. D	72. E	-1.395. D	0.005. D	3.65

Supplementary Table 2. SART3 genetic variants and corresponding details. PolyPhen2 PD = probably damaging. PROVEAN D = deleterious, N = neutral. MuPro D = decreased stability. SIFT D = damaging, T = tolerated. SNAP E= Effect. GnomAD v2.1.1.

Experiment code and facility	Date	Cas9 mRNA or protein conc.	gRNA conc.	Repair template conc.	# zygotes inj.	#2cell	#transf./ #mum	#liveborn	Genotype
**c.2507G>A ; p.Arg836Gln (Exon 17 variant)**									
Sart3-Ex17-A UQ	190314	20ng/ul	10ng/ul	10ng/ul	154	51	2 mums	0	
Sart3-Ex17-B UQ	10514	20ng/ul	10ng/ul	30ng/ul	220	154	2 mums	2 born and eaten	
Sart3-Ex17-C UQ	260514	20ng/ul	10ng/ul	10ng/ul	344	140	7 mums	8 born (3 eaten)	WT
Sart3Ex17-D UQ	240714	20ng/ul	10ng/ul	10ng/ul	251	95	4 mums	2 born	WT
Sart3Ex17-E UQ	310714	20ng/ul	10ng/ul	10ng/ul	358	150	7 mums	4 born	WT
Sart3Ex17 (18mer)	260814	20 ng/ul	10ng/ul	10 ng/ul	402	168	8 mums	4 born	WT
Sart3 Exon 17 UQ	90914	20 ng/ul	10ng/ul	10 ng/ul	247	120	6 mums	6 born	5 WT, 1 with frameshift mutation
Sart3 Exon17 UQ	220914	20 ng/ul	10ng/ul	10 ng/ul	266	106	5 mums	2 born	WT
Sart3Exon17 UQ	21214	10 ng/ul	5 ng/ul	10 ng/ul	292	?	3 mums	6 born but died	
Sart3 R836Q WEHI	51115					155		2 born	2 born but died at birth, both carried indel at target.
**c.631G>A ; p.Glu211Lys (exon 4 variant)**									
exon4 #1 UQ Strategy	3/9/2017	30 ng/μl	15 ng/μl	10 ng/μl	150	110	5 mums	0	
exon4 #2 UQ Strategy	18/5/17	30 ng/μl	15 ng/μl	10 ng/μl	100	50	2 mums	5 born, all died; tissue recovered for 3	2x WT; 1 het with a small insertion
exon4 #3 UQ Strategy	13/7/17	30 ng/μl	15 ng/μl	10 ng/μl	150	130	5 mums	0	
exon4 #4 UQ Strategy	24/8/17	30 ng/μl	15 ng/μl	10 ng/μl	120	90	4 mums	7 born, all died; tissue recovered for 5	3x WT, 2x with indels

Supplementary Table 3. CRISPR editing attempts in mice.

UQ = University of Queensland, WEHI = Walter and Eliza Hall Institute.

Supplementary Data Guide

Supplementary Data 1. Extended clinical phenotype. A table with additional clinical details for the nine affected individuals from six families.

Supplementary Data 2. RNA sequencing and proteomic analysis of iPSCs .
RNA sequencing data: Tab 2. Significantly differentially expressed (DE) RNA-seq genes (FDR<0.05), Tab 3. DAVID analysis for all DE genes, Tab 4. DAVID analysis for subsets of DE genes, Tab 5. Differential Transcript Usage (DTU) analysis. Mass Spectrometry/ proteomic analysis of iPSCs: Tab 6. All significantly differentially expressed proteins, Tab 7.DAVID analysis for DE proteins. Combined RNA-seq and proteomic datasets: Tab 8. Genes that are DE in both RNA and protein datasets in the same direction , Tab 9. DAVID GO analysis of common DE genes/proteins.

Supplementary Data 3. Primer sequences. The nucleotide sequences are provided for all primers used in the study.

Supplementary Data 4. Antibody details. Company, catalogue number, dilution and species reactivity provided for all antibodies used in the study.

Supplementary References

[1] Tomancak P, Beaton A, Weiszmann R, Kwan E, Shu S, Lewis SE, Richards S, Ashburner M, Hartenstein V, Celniker SE, Rubin GM. Systematic determination of patterns of gene expression during Drosophila embryogenesis. Genome Biol. 2002;3(12):RESEARCH0088. doi: 10.1186/gb-2002-3-12-research0088. Epub 2002 Dec 23. PMID: 12537577; PMCID: PMC151190.

[2] Tomancak P, Berman BP, Beaton A, Weiszmann R, Kwan E, Hartenstein V, Celniker SE, Rubin GM. Global analysis of patterns of gene expression during Drosophila embryogenesis. Genome Biol. 2007;8(7):R145. doi: 10.1186/gb-2007-8-7-r145. PMID: 17645804; PMCID: PMC2323238.

[3] Hammonds AS, Bristow CA, Fisher WW, Weiszmann R, Wu S, Hartenstein V, Kellis M, Yu B, Frise E, Celniker SE. Spatial expression of transcription factors in Drosophila embryonic organ development. Genome Biol. 2013 Dec 20;14(12):R140. doi: 10.1186/gb-2013-14-12-r140. PMID: 24359758; PMCID: PMC4053779.

[4] Drysdale R; FlyBase Consortium. FlyBase : a database for the Drosophila research community. Methods Mol Biol. 2008;420:45-59. doi: 10.1007/978-1-59745-583-1_3. PMID: 18641940.

[5] Larkin A, Marygold SJ, Antonazzo G, Attrill H, Dos Santos G, Garapati PV, Goodman JL, Gramates LS, Millburn G, Strelets VB, Tabone CJ, Thurmond J; FlyBase Consortium. FlyBase: updates to the Drosophila melanogaster knowledge base. Nucleic Acids Res. 2021

Jan 8;49(D1):D899-D907. doi: 10.1093/nar/gkaa1026. PMID: 33219682; PMCID: PMC7779046.
