# Supplementary material for: Variants in SART3 cause a spliceosomopathy characterised by failure of testis development and neuronal defects
Source: Nat Commun. 2023 Jun 9;14:3403. doi: 10.1038/s41467-023-39040-0 (PMC10256788; doi:10.1038/s41467-023-39040-0)
Supplement: Supplementary file 3 — Description of Additional Supplementary Files [file 41467_2023_39040_MOESM3_ESM.pdf]

File name: Supplementary Data 1

Description: Extended clinical phenotype. A table with additional clinical details for the nine affected individuals from six families.

File name: Supplementary Data 2

Description: RNA sequencing and proteomic analysis of iPSCs. RNA sequencing data: Tab 2. Significantly differentially expressed (DE) RNA-seq genes (FDR<0.05), Tab 3. DAVID analysis for all DE genes, Tab 4. DAVID analysis for subsets of DE genes, Tab 5. Differential Transcript Usage (DTU) analysis. Mass Spectrometry/proteomic analysis of iPSCs: Tab 6. All significantly differentially expressed proteins, Tab 7. DAVID analysis for DE proteins. Combined RNA-seq and proteomic datasets: Tab 8. Genes that are DE in both RNA and protein datasets in the same direction, Tab 9. DAVID GO analysis of common DE genes/proteins.

File name: Supplementary Data 3

Description: Primer sequences. The nucleotide sequences are provided for all primers used in the study.

File name: Supplementary Data 4

Description: Antibody details. Company, catalogue number, dilution and species reactivity provided for all antibodies used in the study.
