# Supplementary material for: Variants in SART3 cause a spliceosomopathy characterised by failure of testis development and neuronal defects
Source: Nat Commun. 2023 Jun 9;14:3403. doi: 10.1038/s41467-023-39040-0 (PMC10256788; doi:10.1038/s41467-023-39040-0)
Supplement: Supplementary file 8 — Reporting Summary [file 41467_2023_39040_MOESM8_ESM.pdf]

nature portfolio

Corresponding author(s): Dr Katie L. Ayers

Last updated by author(s): May 15, 2023

Reporting Summary

Nature Portfolio wishes to improve the reproducibility of the work that we publish. This form provides structure for consistency and transparency in reporting. For further information on Nature Portfolio policies, see our Editorial Policies and the Editorial Policy Checklist.

Statistics

For all statistical analyses, confirm that the following items are present in the figure legend, table legend, main text, or Methods section.

n/a	Confirmed	
☐	☒	The exact sample size (*n*) for each experimental group/condition, given as a discrete number and unit of measurement
☐	☒	A statement on whether measurements were taken from distinct samples or whether the same sample was measured repeatedly
☐	☒	The statistical test(s) used AND whether they are one- or two-sided<br>*Only common tests should be described solely by name; describe more complex techniques in the Methods section.*
☐	☒	A description of all covariates tested
☐	☒	A description of any assumptions or corrections, such as tests of normality and adjustment for multiple comparisons
☐	☒	A full description of the statistical parameters including central tendency (e.g. means) or other basic estimates (e.g. regression coefficient) AND variation (e.g. standard deviation) or associated estimates of uncertainty (e.g. confidence intervals)
☐	☒	For null hypothesis testing, the test statistic (e.g. *F*, *t*, *r*) with confidence intervals, effect sizes, degrees of freedom and *P* value noted<br>*Give P values as exact values whenever suitable.*
☒	☐	For Bayesian analysis, information on the choice of priors and Markov chain Monte Carlo settings
☒	☐	For hierarchical and complex designs, identification of the appropriate level for tests and full reporting of outcomes
☒	☐	Estimates of effect sizes (e.g. Cohen's *d*, Pearson's *r*), indicating how they were calculated

Our web collection on statistics for biologists contains articles on many of the points above.

Software and code

Policy information about availability of computer code

Data collection	WES data collection ISR1/ISR2 - Illumina TruSeq and Illumina HiSeq 2000; TUN1 Agilent SureSelect Human All Exon V4 and Illumina HiSeq2000 platform with TruSeq v3 chemistry. ISR3 Novaseq6000 platform (Illumina) using the IDT_xGen_Exome_Research_Panel_v2 kit (IDT). ITA1 SureSelectQXT Clinical Research Exome V2 (Agilent Technologies) and run on a NextSeq500 sequencer (Illumina). FRA1 SeqCap EZ MedExome capture kit (Roche) and sequencing on Illumina NextSeq 500.<br>Western blots data were collected on the Amersham Imager 680, and analysis carried out using the Amersham Imager 680 integrated software v2.0.0.<br>qRT-PCR data were collected on the Roche Lightcycler 480 II system, using the LC480 analysis software v1.5.1.62.<br>Imaging data were collected using the ZEISS ZEN Black software (v2.3 SPI) (Zeiss Microscopy, Thornwood, NY) software installed on a ZEISS LSM 780 confocal microscope. RNA sequencing data were collected with the NovaSeq 6000 system (Illumina, San Diego, California). RT-PCR data were collected using the TapeStation 2200 Instrument and Dl000 screen-tapes (Agilent Technology). MS data were collected on a QExactive Plus mass spectrometer (Thermo Fisher Scientific).
Data analysis	WES analysis (ISR1 and ISR2 families) used the following programs used Novoalign v2.07.17, MarkDuplicates from Picard (http://picard.sourceforge.net/), SAMtools v0.1.18, ANNOVAR 28 Nov 2011 version.<br>Estimation of relatedness used the genome analysis option of PLINK, MERLIN,<br>WES of TUN1 used GATK v1.6 and Picard v1.62, SAMtools v0.1.18. EnsEMBL SNP Effect Predictor (http://www.ensembl.org/homosapiens/userdata/uploadvariations)<br>WES of ISR3 used Genoox data analysis platform.<br>WES of ITA1 used BWA v0.7.5, Genexy software (https://geneyx.com),<br>WES of FRA1 used the BaseSpace cloud computing platform (with BWA 2.1 and GATK Unified Genotyper 1.6) and the VariantStudio v.3.0 software provided by Illumina.

Western blot analysis was carried out using the GE Amersham Imager 680 analysis tool v2.0.0.
TapeStation Analysis Software A.01.05 (SRl) was used to analyse RT-PCR data.
SART3 protein modelling used the ClustalX program v2.1 and crystal structures were visualised in the RCSB PDB 3D protein viewer (Mol*Plugin 3.29.0).
Graphpad Prism v9 was used for statistics and to plot graphs for qRT-PCR, RT-PCR, western blot data, organoid size analyses.
RNA sequencing analysis used the following tools: STAR v2.5.2, PicardTools v2.0.1, featureCounts v1.5.0, edgeR v3.34.1 . DTU analysis used salmon and SatuRn (v1.0.0). The Database for Annotation, Visualisation and integrated Discovery (DAVID; 2021 update) was used for KEGG pathway analysis or Gene Ontology (biological pathway) enrichment analysis. Integrated Genomics Viewer (IGV) v2.12.3 was used to visualise RNA-seq reads.
Mass spectrometric raw files were analyzed with MaxQuant vl.6.5.0. Downstream statistical analysis was performed in Perseus vl.6.2.3 and LFQ-Analyst.

For manuscripts utilizing custom algorithms or software that are central to the research but not yet described in published literature, software must be made available to editors and reviewers. We strongly encourage code deposition in a community repository (e.g. GitHub). See the Nature Portfolio guidelines for submitting code & software for further information.

Data

Policy information about availability of data

All manuscripts must include a data availability statement. This statement should provide the following information, where applicable:

- Accession codes, unique identifiers, or web links for publicly available datasets
- A description of any restrictions on data availability
- For clinical datasets or third party data, please ensure that the statement adheres to our policy

The MS proteomic data generated in this study have been deposited in the ProteomeXchange Consortium via the PRIDE89 partner repository under accession code PXD032816. RNA-seq data generated in this study have been deposited in the Sequence Read Archive (SRA) database under bioproject accession code PRJNA886829. SART3 variants have been entered into ClinVar (SCV003842293 - SCV003842300). Exome sequencing data can be requested by contacting the corresponding author, with a response within 1 month. Due to ethics restrictions on storing and sharing our paediatric patient exome data, this data will have controlled access and will be limited to individuals who enter a research agreement. Use of this genomic data will be restricted to those named on the agreement, and exome data will be patient de-identified. The following databases were used; human reference genomes hg19 and hg38, Gencode human annotation release 40 (GRCh37), dbSNP 132, dbSNP 138, 1000 Genomes Project, NHLBI Go Exome Sequencing Project, gnomAD, TOPMed, ALFA, Flybase. Source data are provided with this paper.

Human research participants

Policy information about studies involving human research participants and Sex and Gender in Research.

Reporting on sex and gender	Patients were recruited following identification of recessive variants in SART3 as part of clinical or research genomic testing. Both female and male patients and family members were recruited and sex was determined by sex-chromosome complement, whilst gender was self reported. In cases where patients were unable to self report gender, it was based on clinical observations of external genitalia.
Population characteristics	The study included patients born with a congenital disorder include neurodevelopmental defects and 46,XY gonadal dysgenesis, in whom a variant in the SART3 gene had been identified via genomic sequencing, and their families. Those recruited to the study were infants or children, and their families (parents and siblings) ranged in age.
Recruitment	Patients were recruited by their treating clinicians following identification of recessive variants in SART3 as part of clinical or research genomic testing. Most probands were recruited as infants or children during the course of treatment for their condition. The ages at clinical assessment are provided in the detailed clinical notes. Affected children with both female, ambiguous, and male sexual characteristics were recruited (and chromosomal sex was later confirmed). Female and male family members including parents and siblings were also recruited, at any age.
Ethics oversight	This research complies with all relevant ethical regulations and was approved by the following boards/committees: Human Research Ethics Committee, Royal Children's Hospital, Melbourne Australia (HREC22073). French Ethical Committee (2014/18NICB; registration no. IRB00003835), Bambino Gesù Children's Hospital (registration no. 1779_OPBG_2019), Assistance Publique Hôpitaux Marseille (reference PADS21-282). Written informed consent for participation in the study was obtained from all human research participants (or their guardians). All parents/guardians have seen and consented to the research and to publication of data within the context of the paper. Human foetal testis tissue was obtained following elective termination of pregnancy during the 1st trimester at the Departments of Gynaecology at Copenhagen University Hospital (Rigshospitalet) and Hvidovre Hospital, Denmark, following informed written and oral consent (ethics permit H-1-2012-007). None of the terminations were for fetal or pregnancy pathology. Drosophila work was carried out according to protocols approved by the University of Melbourne Institutional Biosafety Committee (IBC) reference no: 2017/023. Mouse work was conducted according to protocols approved by the University of Queensland Ethics Committee (IMB/435/13/NHMRC/ARC; IMB/445/12/NHMRC/BREED; IMB/232/13/NHMRC/ARC).

Note that full information on the approval of the study protocol must also be provided in the manuscript.

Field-specific reporting

Please select the one below that is the best fit for your research. If you are not sure, read the appropriate sections before making your selection.

☒ Life sciences ☐ Behavioural & social sciences ☐ Ecological, evolutionary & environmental sciences

For a reference copy of the document with all sections, see nature.com/documents/nr-reporting-summary-flat.pdf

Life sciences study design

All studies must disclose on these points even when the disclosure is negative.

Sample size	In general, sample sizes were based on even group distribution and no statistical method was used to predetermine sample size owing to the exploratory nature of the study. For RNA sequencing, sample size was chosen based on previously published RNA-seq papers using cell lines and the sample size needed to perform statistical tests. qPCR analyses: sample size was chosen based on previously published papers using qPCR analysis to compare gene expression in siRNA mediated knockdown. For fly studies, sample sizes were chosen based on published work analysis phenotypes associated with novel genes.
Data exclusions	Minimal data were excluded from the publication. For qRT-PCR a technical replicate or sample was excluded if amplification failed. For neuronal differentiation, data was not collected for differentiations where homozygous SART3 variant cells failed to produce sufficient numbers of neurons to analyse.
Replication	Experiments were repeated independently yielding similar results the following number of times: Immunofluorescent staining of endogenous or transiently transfected WT or variant SART3 in HEK293t cells - three independent experiments with both SART3 and FLAG antibodies. Drosophila crosses and analyses - twice with each RNAi line. For tj-Gal4 crosses, these were carried out once with the dj-GFP reporter and twice without reporter for each RNAi line. Staining of human fetal testes and ovaries - once (on two different tissue samples per sex). Repeats were not possible due to scarcity of tissue. Staining and RNA analysis of gonadal organoid differentiation - three independent differentiation experiments were carried out with three biological replicate monolayers/organoids analysed for each cell line at each timepoint. Neuronal staining experiments - five independent differentiation experiments were analysed, with only three providing sufficient numbers of homozygous variant neurons to analyse. Staining for MAP2, BIII, SART3 and CC3 - three independent differentiations analysed, staining for ANK-G and Neurofilament - two. Western blotting for SART3 in iPSCs - three independent experiments. Immunofluorescent staining of iPSCs for SART3 and OCT4 – two independent experiments. Embryoid body experiments – two independent experiments. NT2/D1 KD experiments were replicated independently four times. For all other experiments where statistics were derived, details of the repeats, sample number and statistical methods can be found in the Figure Legends.
Randomization	Assortment of organoids, differentiated neurons or fly tissues of equivalent morphology into analysis groups (i.e. different staining or RNA-analysis) was randomized for all experiments.
Blinding	Investigators were not blinded to allocation during analyses and outcome assessment owing to the automated processed used to collect and analyse results.

Reporting for specific materials, systems and methods

We require information from authors about some types of materials, experimental systems and methods used in many studies. Here, indicate whether each material, system or method listed is relevant to your study. If you are not sure if a list item applies to your research, read the appropriate section before selecting a response.

Materials & experimental systems

n/a	Involved in the study
☐	☒ Antibodies
☐	☒ Eukaryotic cell lines
☒	☐ Palaeontology and archaeology
☐	☒ Animals and other organisms
☒	☐ Clinical data
☒	☐ Dual use research of concern

Methods

n/a	Involved in the study
☒	☐ ChIP-seq
☒	☐ Flow cytometry
☒	☐ MRI-based neuroimaging

Antibodies

Antibodies used	Sigma Rabbit Anti-FLAG F7425 Lot 086M4803V. Western blotting (1in10000) and IF (1in5000)<br>Abcam Mouse Anti-SART3 ab84671 Lot GR281975-3. Western blotting (1in1500) and IF (1in300)<br>Abcam Rabbit Anti-Beta Tubulin-HRP ab21058. Western blotting (1in10000)<br>BD Bioscience Mouse Anti-OCT3/4 611202 Lot 9727. Western blotting (1in500)<br>Santa Cruz Mouse Anti-AASS sc-365417 Lot F2711. Western blotting (1in1000)<br>Abnova Mouse Anti-SART3 H00009733-B01P. IF (1in300)

Abcam Chicken Anti-MAP2 ab5392. IF (1in5000)
Santa Cruz Goat Anti-SOX17 sc-17355. IF (1in50)
Agilent DAKO Mouse Anti-SMA M0851. IF (1in25)
BioLegend Mouse Anti-TRA-1-81-A647 330706. Flow cytometry (1in100)
BioLegend Mouse Anti-SSEA4 330408. Flow cytometry (1in100)
BD Bioscience Mouse Anti-CD9-FITC 555371. Flow cytometry (1in20)
BioLegend Mouse Anti-EPCAM-PeCy7 324222. Flow cytometry (1in100)
Santa Cruz Mouse Anti-AMH sc166752 Lot B0617. IF (1in300)
Santa Cruz Goat Anti-OCT3/4 sc-8629 Lot G110. IF (1in600)
Novus Goat Anti-FOXL2 NB100-1277 Lot S3. IF (1in200)
BD Bioscience Mouse Anti-Ki67 bd550609. IF (1in200)
Biolegend Rabbit Anti-Neurofilament 841001. IF (1in500)
Santa Cruz Rabbit Anti-Ankryn-G sc28561. IF (1in500)
Sigma Mouse Anti-TUJ1 MAB1637. IF (1in2000)
Millipore Guinea Pig Anti-NeuN ABN90. IF (1in500)
Santa Cruz Mouse Anti-GATA4 sc-25310 Lot C1417. IF (1in400)
R & D systems Goat Anti-SOX9 AF3075. IF (1in300)
Abcam Rabbit Anti-COLIV ab6586 Lot GR3278425-1. IF (1in300)
Cell Signalling Rabbit Anti-Cleaved Caspase 3 9930 Lot 23. IF (1in1000)
DSHB Mouse Anti-Futsch 22C10. IF (1in100)
DSHB Rat Anti-Elav 7E8A10. IF (1in100)
Abcam Chicken Anti-GFP ab13970. IF (1in2000)
DSHB Mouse Anti-Axons BP102. IF (1in50)
Santa Cruz Goat Anti-Vasa DC13. IF (1in50)
DSHB Mouse Anti-FasIII 7G10. IF (1in50)

Validation

Sigma Rabbit Anti-FLAG F7425: Validation performed by manufaturers includes immunoblotting with whole extract HEK-293T cells overexpressing both C-terminal and N-terminal Flag tagged proteins which confirmed a specific band at correct size (https://www.sigmaaldrich.com/AU/en/product/sigma/f7425). Antibody specificity was also validated in our lab in western blot and IF, where no staining or band was observed in the absence of FLAG-epitope (i.e. cells transfected with an empty vector control).

Abcam Mouse Anti-SART3 ab84671: Validated performed by Abcam in immunohistochemistry by showing specific staining in Formalin-Fixed Paraffin-Embedded Human pancreatic islet cell tumor tissue in 1in100 and 1in250 (https://www.abcam.com/products/primary-antibodies/sart3-antibody-ab84671.html). The specificity of antibodies from Abcam was confirmed by the manufacturer via analyzing cells that either do or do not express the target protein within the same tissue and in different tissues, using multi-normal human tissue microarrays (TMAs), resulting in the analysis of many tissues at the same time, providing uniformity as all tissues are exposed to the exact same conditions, as stated on their website (https://www.abcam.com/primary-antibodies/how-we-validate-our-antibodies).In our lab, this antibody showed specificity to transiently transfected SART3 in western blots and IF, and intensity was reduced in western blotting in SART3 variant cells and SART3 siRNA transfected cells.

Abcam Rabbit Anti-Beta Tubulin-HRP ab21058: The specificity of antibodies from Abcam has been confirmed by the manufacturer via analyzing cells that either do or do not express the target protein within the same tissue and in different tissues, using multi-normal human tissue microarrays (TMAs), resulting in the analysis of many tissues at the same time, providing uniformity as all tissues are exposed to the exact same conditions, as stated on their website (https://www.abcam.com/primary-antibodies/how-we-validate-our-antibodies). Specifically, this antibody has been validated by Abcam for both WB and IHC-P has been carried out (see https://www.abcam.com/products/primary-antibodies/hrp-beta-tubulin-antibody-loading-control-ab21058.html). Validation in immunohistochemistry in Formalin-Fixed Paraffin-Embedded normal human colon tissue showed specific staining. The antibody detects a single band of approximately 50 kDa (predicted molecular weight: 50 kDa) in both HeLa and A431 (Human epidermoid carcinoma) whole cell lysates, and in our hands Hek293t cells and iPSCs.

BD Bioscience Mouse Anti-OCT3/4 611202 has been validated in western blot analysis in both human and mouse ES cell lines which express high levels of this protein. Lysates from H9 human ES cells and ES-E14TG2a mouse ES cells (ATCC CRL-1821) and were probed with antibody at titrations of 2.0, 1.0, and 0.5 µg/ml showing a specific band of 46 kDa (see https://www.bdbiosciences.com/en-au/products/reagents/microscopy-imaging-reagents/immunofluorescence-reagents/purified-mouse-anti-oct3-4.611203). In our hands, immunofluorescent staining of human iPSCs and human fetal gonadal germ cells also showed specific nuclear staining which is not present in other cells or differentiated tissues.

Santa Cruz Mouse Anti-AASS sc-365417 antibody has been validated by Santa Cruz for use in Western Blot analysis in both AN3 CA Cell Lysate: sc-24662 (human endometrial adenocarcinoma cells) and HEK293 (Human embryonic kidney cell line), where it shows a clean single band at 116-128 kDa. https://datasheets.scbt.com/sc-365417.pdf. Please refer to this website for citations.

Abnova Mouse Anti-SART3 H00009733-B01P has been validated by the manufacturer, Abnova, in Western Blot analysis to show SART3 expression in human pancreas tissues where it recognized a single band at the correct size. In Western Blot analysis of SART3 expression in transfected HEK293T cell lines carried out by manufacturer and us shows SART3 at just one size, and this is increased in transfected cells showing specificity. Additional validation of the antibody: immunofluorescence of purified MaxPab antibody to SART3 on HeLa cell. [see website for validation details and citations. https://www.abnova.com/products/products_detail.asp?catalog_id=H00009733-B01

Abcam Chicken Anti-MAP2 ab5392 has been validated by Abcam, who have confirmed speficity in immunocytochemistry and Western Blot analysis in mouse and rat tissue. it has been extensively referenced in the literature

Santa Cruz Goat Anti-SOX17. In our hands this antibody stained only a neuronal population in embryoid bodies, with no additional staining. Please see manufacturers website for other relevant citations (https://www.scbt.com/p/sox-17-antibody-s-20). In Li et al, Cell Death and Disease 2018, specificity of this antibody in human cells was demonstrated by using transient transfection of SOX17 into HeLa cells. In de Jong et al, J. Pathology 2008 western blotting shows SOX17 expression with this antibody specific to inTCam-2 and no SOX17 expression in the EC cell lines NTera-2, NCCIT, and 833KE

The mouse anti-human SMA antibody has been validated by Agilent-DAKO and has been optimized for immunohistochemistry. The antibody is used clinically for classification of smooth muscle cells, myofibroblasts, and myoepithelial cells and their tumors such as leiomyoma and leiomyosarcoma (see https://www.agilent.com/en/product/immunohistochemistry/antibodies-controls/primary-antibodies/actin-%28smooth-muscle%29-%28concentrate%29-76542). In SDS-PAGE immunoblotting of the a-smooth muscle isoform of actin, the antibody labels a band corresponding to a-smooth muscle actin.

BioLegend have verified that the anti-TRA-1-81 A647 330706 antibody is reactivity to human samples. It has been verified for Flow cytometric analysis of antibody surface-stained cells, and quality controlled Immunocytochemistry. Each lot of this antibody is quality control tested by immunofluorescent staining with flow cytometric analysis. see website for all application citations https://www.biolegend.com/nl-be/products/alexa-fluor-647-anti-human-tra-1-81-antibody-4827

BioLegend have verified that the Mouse Anti-SSEA4 330408 antibody is reactivity to human samples. It has been verified for Flow cytometric analysis of antibody surface-stained cells, and quality controlled Immunocytochemistry. For example in NCCIT (human teratocarcinoma cell line) stained with MC-813-70 Alexa Fluor® 647. Each lot of this antibody is quality control tested by immunofluorescent staining with flow cytometric analysis. see website for all application citations https://www.biolegend.com/nl-be/products/alexa-fluor-647-anti-human-ssea-4-antibody-4823

BD Bioscience Mouse Anti-CD9-FITC 555371 has been shown to have reactivity in Human (QC Testing). Application: Flow cytometry (Routinely Tested). see manufacturers website for details https://www.bdbiosciences.com/en-au/products/reagents/flow-cytometry-reagents/research-reagents/single-color-antibodies-ruo/fitc-mouse-anti-human-cd9.555371

BioLegend Mouse Anti-EPCAM-PeCy7 324222 was quality tested for flow cytometric analysis by BioLegend. see https://www.biolegend.com/nl-be/products/pe-cyanine7-anti-human-cd326-epcam-antibody-8107 for full details and all citations

Santa Cruz Mouse Anti-AMH sc166752 is recommended for detection of precursor and mature MIS of mouse, rat and human origin by Western Blotting, immunoprecipitation, immunofluorescenceand solid phase ELISA. Santa Cruz has validated MIS Antibody (B-11): sc-166752 in Western blot analysis of MIS expression in non-transfected: sc-110760 (A) and human MIS transfected: sc-111265 (B) 293 whole cell lysates, and observed a clear band at correct Molecular Weight of MIS: 70/74 kDa. (https://www.scbt.com/p/mis-antibody-b-11)

Santa Cruz Goat Anti-OCT3/4 sc-8629 has been validated by manufacturers and used in many citations to mark pluripotent cells. In our hands this antibody stained only pluripotent stem cell and germ cells with a specific nuclear staining and did not stain surrounding tissues or cells. Please see website for relevant citations https://www.scbt.com/p/oct-3-4-antibody-c-20

Novus Goat Anti-FOXL2 NB100-1277 has been validated by the manufacturers for western blot and IF applications https://www.novusbio.com/products/foxl2-antibody_nb100-1277#datasheet. In western blot, it detects a 50kDa band specifically in cells of ovarian origin. In our hands, it specifically stained ovarian sections and not testicular sections.

BD Bioscience has validated Mouse Anti-Ki67 bd550609 for immunohistochemistry, showing specific staining of Ki-67 positive cells (proliferating cells) in formalin-fixed paraffin embedded sections of normal human tonsil were reacted with the anti-Ki-67 antibody. In our hands it specifically marked the gonadal germ cells and no other cells in the surrounding tissue.

Biolegend has verified Rabbit Anti-Neurofilament 841001 reactivity in human, rat and mouse. The antibody is verified for use in Western Blot and quality tested for immunohistochemistry. Each lot of this antibody is quality control tested by formalin-fixed paraffin-embedded immunohistochemical staining, the manufacturer's have illistrated the specificity on formalin fixed paraffin embedded human cortex tissue. (https://www.biolegend.com/en-us/products/anti-neurofilament-m-nf-m-antibody-11130). In our hands this antibody specifically stained differentiated neurons.

Santa Cruz have validated Rabbit Anti-Ankryn-G sc28561 for use in several applications and it has been used in numerous publications as a specific marker of the neuronal axon initial segment.Yoon et al, Molecular Psychiatry 2021 demonstrated specificity of this antibody to Ankyrin-G via immunoblotting and immunoprecipitation experiements and via IF on neurons. Please see website for full citation list and validation details https://www.scbt.com/p/ankyrin-g-antibody-h-215

Sigma Mouse Anti-TUJ1 MAB1637. The manufacturers have validated reactivity in brain tissue of rat, mouse, avian, human, bovine, monkey, pig, sheep, and routinely evaluate the antibody by Western Blot on Mouse Brain lysates. Reacts speicifically with the C-terminus of the neuron specific beta III isoform of tubulin - Positive control: Brain tissue or neurons of any type. Negative control: Non neural tissue or Astrocytes. In our hands, this antibody only stained differentiated neurons.

Millipore Guinea Pig Anti-NeuN ABN90 has been tested by the manufacturer for use in Immunohistochemistry and Western Blotting. Western Blotting in E16 Mouse brain tissue lysate showed specificity for target protein. In our hands this antibody only stained differentiated neurons.

Santa Cruz Mouse Anti-GATA4 sc-25310 has been verified by Santa Cruz Direct in immunoperoxidase staining of fixed tissues/cells, showing nuclear localisation in sample types: human colon, HeLa cells, human ovary tissue showing nuclear staining of follicle cells and ovarian stroma cells. It has been validated in western blot analysis of GATA-4 expression in NIH/3T3 (mouse) nuclear extract, and Neuro-2A (mouse), ATL-16T (human) and AMJ2-C8 (mouse) whole cell lysates. (see website https://www.scbt.com/p/gata-4-antibody-g-4)

R & D systems have validated their Goat Anti-SOX9 AF3075 antibody for use in both western blot analysis and immunocytochemistry. The manufaturer's have demonstrated specific detection of SOX9 in a range of human cells lines; for immunocytochemistry: HEK293 Human Cell Line and SOX9 in BG01V Human Embryonic Stem Cells. Specific staining was localized to nuclei. For Western Blot analysis: KATO-III human gastric carcinoma cell line, COLO 205 human colorectal adenocarcinoma cell line, and Hep3B human hepatocellular carcinoma cell line. A specific band was detected for SOX9 at approximately 75 kDa (see https://www.rndsystems.com/products/human-sox9-antibody_af3075). In our hands, SOX9 specifically detects Sertoli cells in male gonad tissue only.

Abcam has verified Rabbit Anti-COLIV ab6586in reactivity in human, among other mammals and can be used in Western-blot analysis and a range of staining procedures. Abcam have validated the anibody for use in immunohistochemical analysis in human tissue such as Paraffin-embedded human kidney tissue which showed strong staining in glomeruli. Human liver tissue which showed strong specificity to the sinusoids. (see https://www.abcam.com/products/primary-antibodies/collagen-iv-antibody-ab6586.html#lb) The antibody has been referenced in numerous peer-reviewed publications

Validation by Cell Signalling of Rabbit Anti-Cleaved Caspase 3 9930 include western blot analysis showing that this antibody specifically detects cleaved caspase 3 in protein from Jurkat cells treated with Cytochrome C . They also provide western blot analysis of various cell lines, untreated or treated with Staurosporine or with Etoposide which cause apoptosis. The cleaved Caspase-3 antibody showed specific staining in treated cells. MCF7 cells are negative for caspase-3 expression.

DSHB Mouse Anti-Futsch 22C10 has been validated as specific for neuronal poulations in Fujita et al PNAS 1982 and Zipursky et al cell 1984. In Hummel et al Neuron 2000 it was shown that staining disapeared in a Futsch mutant embryo thus demonstrating specificity. See manufacterers website for additional citations https://dshb.biology.uiowa.edu/22C10

DSHB Rat Anti-Elav 7E8A10 has been validated in several publications including Wei et al, Mol Cell 2020, where specificity of the antibody was validated in Drosophila embryos using IF in control and Elav mutants - where staining was completely lost in the latter. See manufacterers website for additional citations https://dshb.biology.uiowa.edu/Rat-Elav-7E8A10-anti-elav

Abcam Chicken Anti-GFP ab13970. IF (1in2000): Validation of this antibody to GFP in a number of contexts is availible by manufacturer https://www.abcam.com/products/primary-antibodies/gfp-antibody-ab13970.html#lb. Specificity to GFP was also observed in our hands where no staining was observed in embryos without a GFP reporter.

DSHB Mouse Anti-Axons BP102: As detailed on manufacturer website https://dshb.biology.uiowa.edu/BP-102-anti-CNS-axons, this antibody strongly stains the axons of the CNS with virtually undetectable staining of neuron cell bodies, the PNS, or any other tissues of the embryo. It has been used in over 14 publications and staining is lost when neurons are lost. - see Ivanov et al PNAS 2004. It is a well established marker of the CNS in flies although the epitope is unknown.

Santa Cruz Goat Anti-Vasa DC13 is specific for a well established marker of the germ cell, Vasa. In Drosophila, its specificity has been validated by staining gonads from Vasa mutant flies, where the staining is lost - see Liu et all, Genes and Dev 2009. See website for additional citations. https://www.scbt.com/p/vasa-antibody-dc-13

DSHB Mouse Anti-FasIII 7G10. This antibody is used to specifically mark the hub of the Drosophila testis. Numerous papers have shown that when the hub cells are disrupted or lost, staining by this antibody is lost illustrating its specificity for this cell population. See multiple papers referenced on manufacturers website https://dshb.biology.uiowa.edu/7G10-anti-Fasciclin-III. In addition, it has been reported that this antibody has been used to successfully immunoprecipitate Fasciclin III illustrating its target Wells et al Development 2013.

Eukaryotic cell lines

Policy information about cell lines and Sex and Gender in Research

Cell line source(s)	iPSCs were derived from commercially available human foreskin fibroblasts (American Type Culture Collection number PCS-201-010)<br>The following cell lines were acquired from commercial sources:<br>NT2Dl ATCC® CRL-1973<br>HEK293T ATCC
Authentication	iPSC lines were checked for genomic integrity using SNP arrays, Sanger sequencing confirmed genome targetting where appropriate, and pluripotency was checked using immunofluroscence, FLOW analysis and embryoid body formation and staining. iPSC lines that have a male sex chromosome were used (46,XY) given the 46,XY-specific gonadal dysgenesis observed in patients and modeled in stem cell experiments.<br>HEK293t and NT2/D1 cell lines have not been authenticated as supplied from commercial sources
Mycoplasma contamination	All cell lines tested for Mycoplasma in house and found negative
Commonly misidentified lines (See ICLAC register)	No commonly misidentified lines were used

Animals and other research organisms

Policy information about studies involving animals; ARRIVE guidelines recommended for reporting animal research, and Sex and Gender in Research

Laboratory animals	Drosophila melanogaster, strains:<br>tubulin-Gal4; tubulin-Gal80ts<br>w1118<br>traffic-jam-Gal4<br>traffic-jam-Gal4; don-juan-GFP<br>elav-Gal4, UAS-GFP<br>repo-Gal4, UAS-GFP<br>RNAi for Rnp4f BL58168 (Bloomington Stock Center), V107063 (KK102446; Vienna Resource Center)<br><br>Drosophila crosses were carried out with newly eclosed females (virgin females) and males less than 1 week old. Ages studied for

phenotypes included stage 10 - 16 embryos and adults up to 1 week old.

Mouse strains used for attempted CRISR-Cas9 KI were C57BL/6J, and included male and female adult mice. As no viable mutant offspring were obtained we are not reporting age or sex for these studies.

Wild animals	No wild animals were used in this study
Reporting on sex	Mice: Standard methods were used for transgenic attempts i.e. females were used as donors and surrogates and were mated with males. As no viable mutant homozygous offspring were obtained for analysis we are not reporting age or sex.<br>Drosophila: Embryos - both females and males were analysed at this stage, as they are indistinguishable in early embryonic stages and we were focused on CNS development. Both females and males KD adults were studied for phenotype and fertility and a male specific fertility phenotype was noted. This was confirmed using imaging analysis of the testes, and further analysis of males was carried out using various drivers and antibody combinations.
Field-collected samples	No field collected samples were included in the study
Ethics oversight	Drosophila work: University of Melbourne Institutional Biosafety Committee (IBC) reference no: 2017/023. Mouse work was conducted according to protocols approved by the University of Queensland Ethics Committee (IMB/435/13/NHMRC/ARC; IMB/445/12/NHMRC/BREED; IMB/232/13/NHMRC/ARC).

Note that full information on the approval of the study protocol must also be provided in the manuscript.
